# Supplementary material for: Strength of functional signature correlates with effect size in autism
Source: Genome Med. 2017 Jul 7;9:64. doi: 10.1186/s13073-017-0455-8 (PMC5501949; doi:10.1186/s13073-017-0455-8)
Supplement: Supplementary file 1 — Trend line robustness analysis. Figure S2. Functional convergences null for matched length and multifunctionality controls. Figure S3. Functional convergence correlation/trend distributions for GWAS. Figure S4. Functional convergence correlation/trend distributions for all network connectivity tests and all gene expression tests. Figure S5. Functional convergence correlation/trend distributions for all MSigDB collections. (DOCX 375 kb) [file 13073_2017_455_MOESM1_ESM.docx]

## Supporting information for:

# Strength of functional signature correlates with effect size in autism

### Supplementary Figures


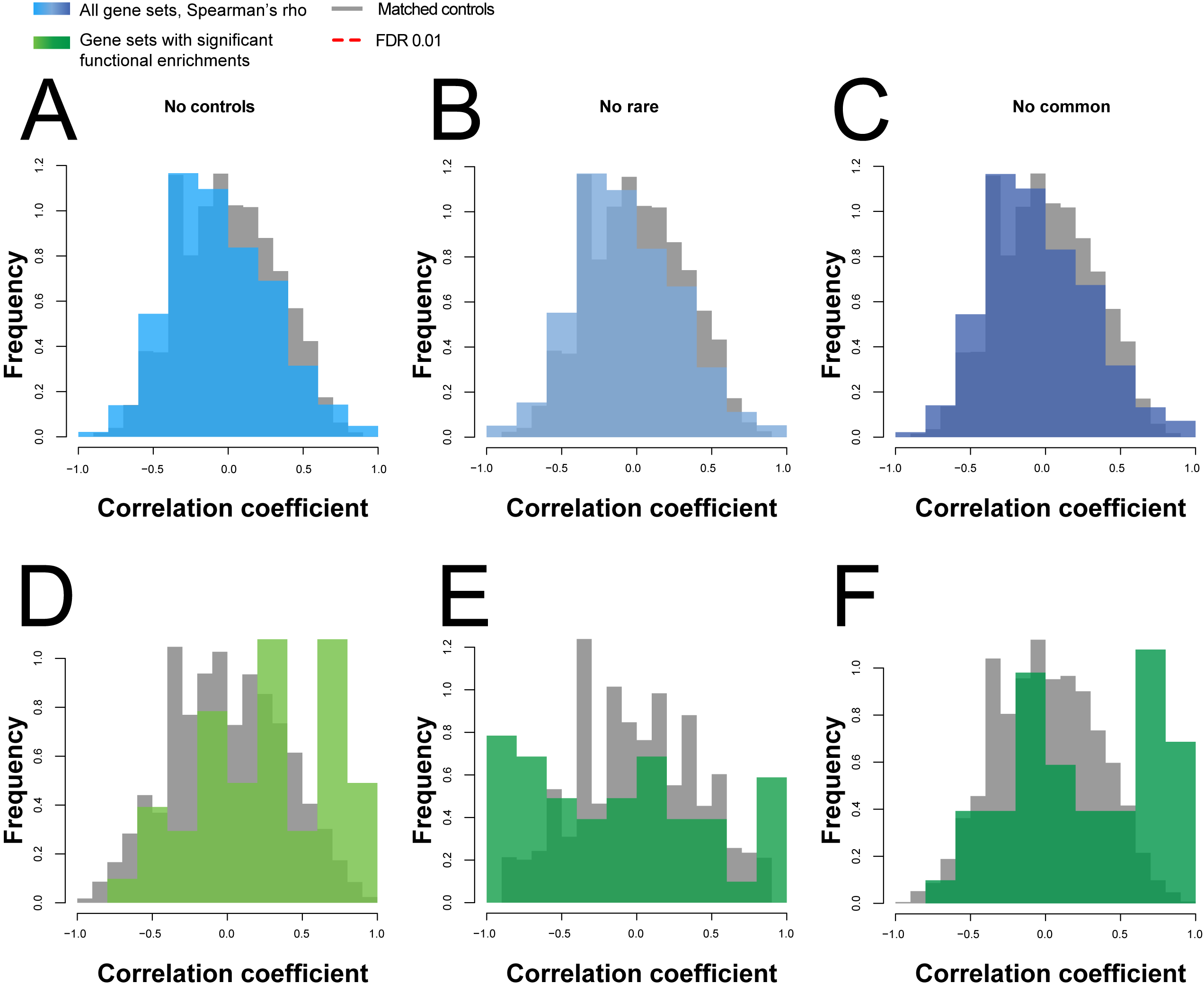


**Fig S1 Trend line robustness analysis.** The functional convergence correlations are dependent on a few of our assumptions. (A) When we remove controls, (B) rare variant gene sets (C) and common gene sets we lose all significant slopes at an FDR of 0.01, and the distributions are still significantly different than the null (Student’s paired T-test *p*<2.2e-16). Looking at the functions with at least one functionally significant gene set, (D) when we remove controls, we still see a shift in distributions towards positive slopes (Student’s paired T-test p~4.2e-7) yet none are significant at a FDR of 0.01. (E) We lose most of the signal when we remove the rare variant gene sets (Student’s paired T-test p~0.02) (F) and the common gene sets even though there is a still a positive trend (Student’s paired T-test p~8.2e-7).


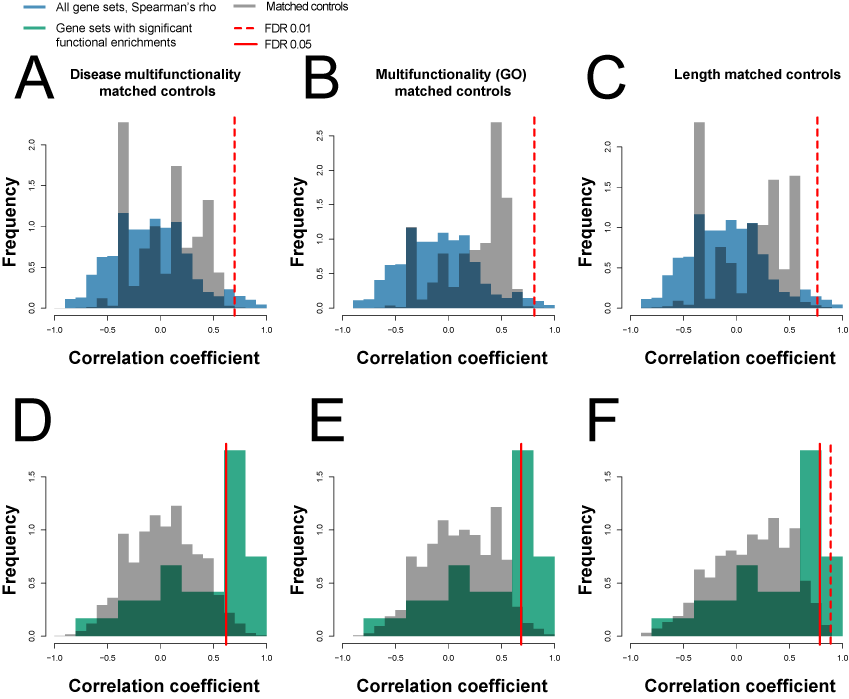


**Fig S2 Functional convergence correlation/trend distribution nulls using matched controls.** (A) For disease matched controls, the distribution of slopes (shown in grey) displays a partial positive trend, yet not as much as the real disease gene sets (Student’s paired T-test p<2.2e-16). (B) The same applies for multifunctional genes (Student’s paired T-test p<2.2e-16) (C) and length matched genes (Student’s paired T-test p<2.2e-16). Using these as nulls, we get many more slopes passing an FDR of 0.01 than the permuted null, with 123, 57 and 87 slopes considered significant. (D) Looking only at the subset of functions that are considered significant (i.e., have at least one gene set collection with a functional signal), the distribution of slopes is almost normal for both the disease (Student’s paired T-test p<2.2e-16) and (E) multifunctional gene matched sets (Student’s paired T-test p<2.2e-16). (F) The length control still has a slight positive trend, but not significant. Although no slopes pass the FDR 0.01 threshold for the multifunctional nulls, there are 3 slopes that pass the FDR 0.01 threshold when using the length matched controls as a null (Student’s paired T-test p<2.2e-16).

**
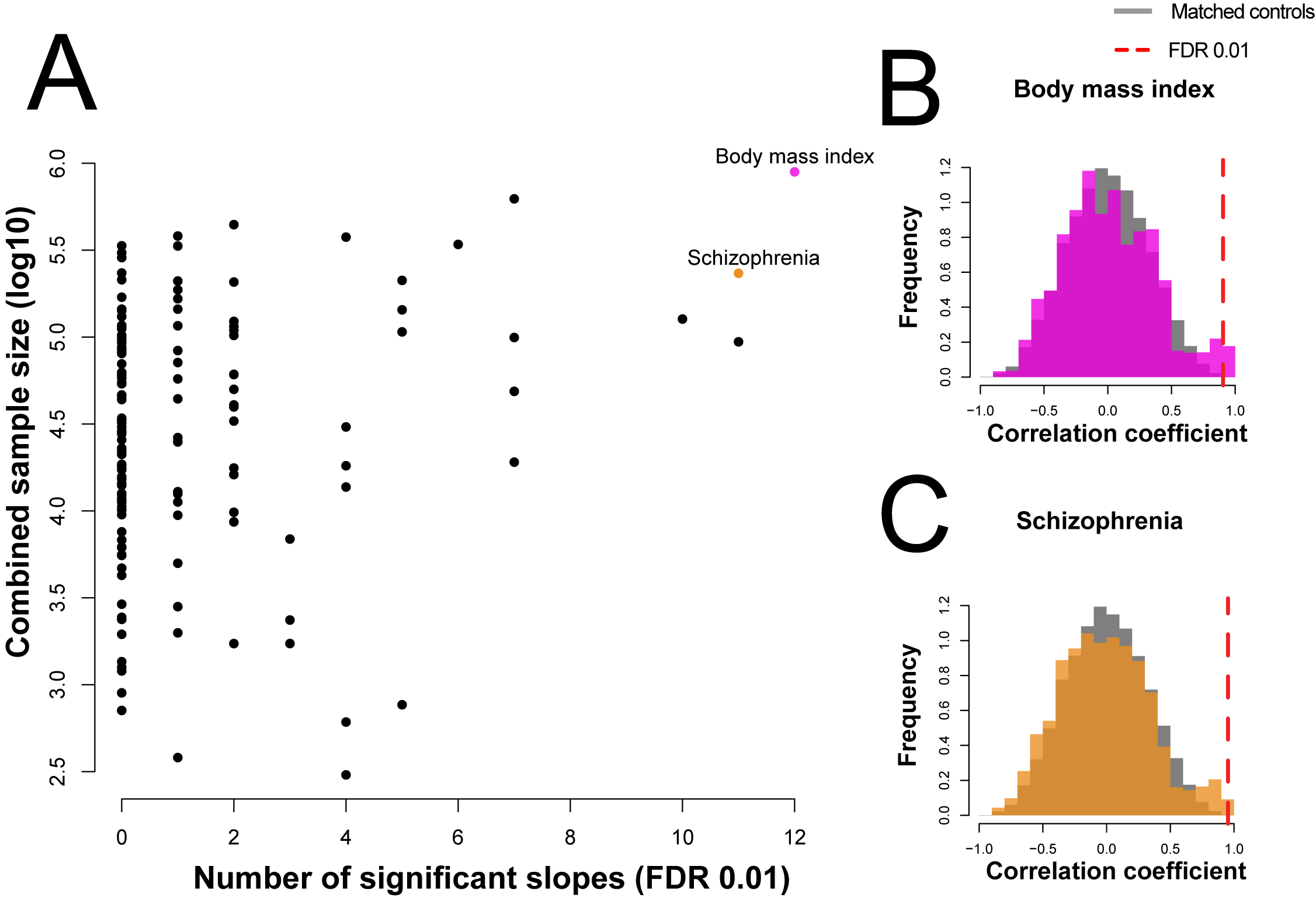
**

**Fig S3 Functional convergence correlation/trend distributions for GWA studies.** (A) Repeating the analysis by substituting the autism GWAs with another GWAs trait or disease, most have no significant slopes in the trend tests, but there is a correlation with the number of samples assessed. All gene sets were downsampled to be comparable. (B) Slope distributions for the BMI GWAs (C) Slope distributions for the schizophrenia GWAs.

**
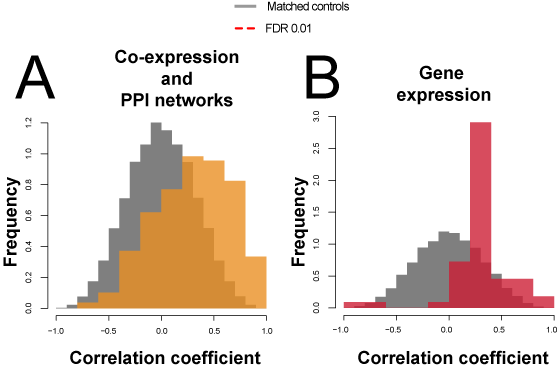
**

**Fig S4 Functional convergence correlation/trend distributions for all network connectivity tests and all gene expression tests.**  We observe slight enrichments over the null but no significantly enriched slopes at an FDR of 0.01. (A) For all network connectivity tests, including both co-expression and extended protein-protein interactions (Student’s paired T-test p~6e-6). (B) For all tissue, sex and population specific gene expression (Student’s paired T-test p<2.2e-16).

**
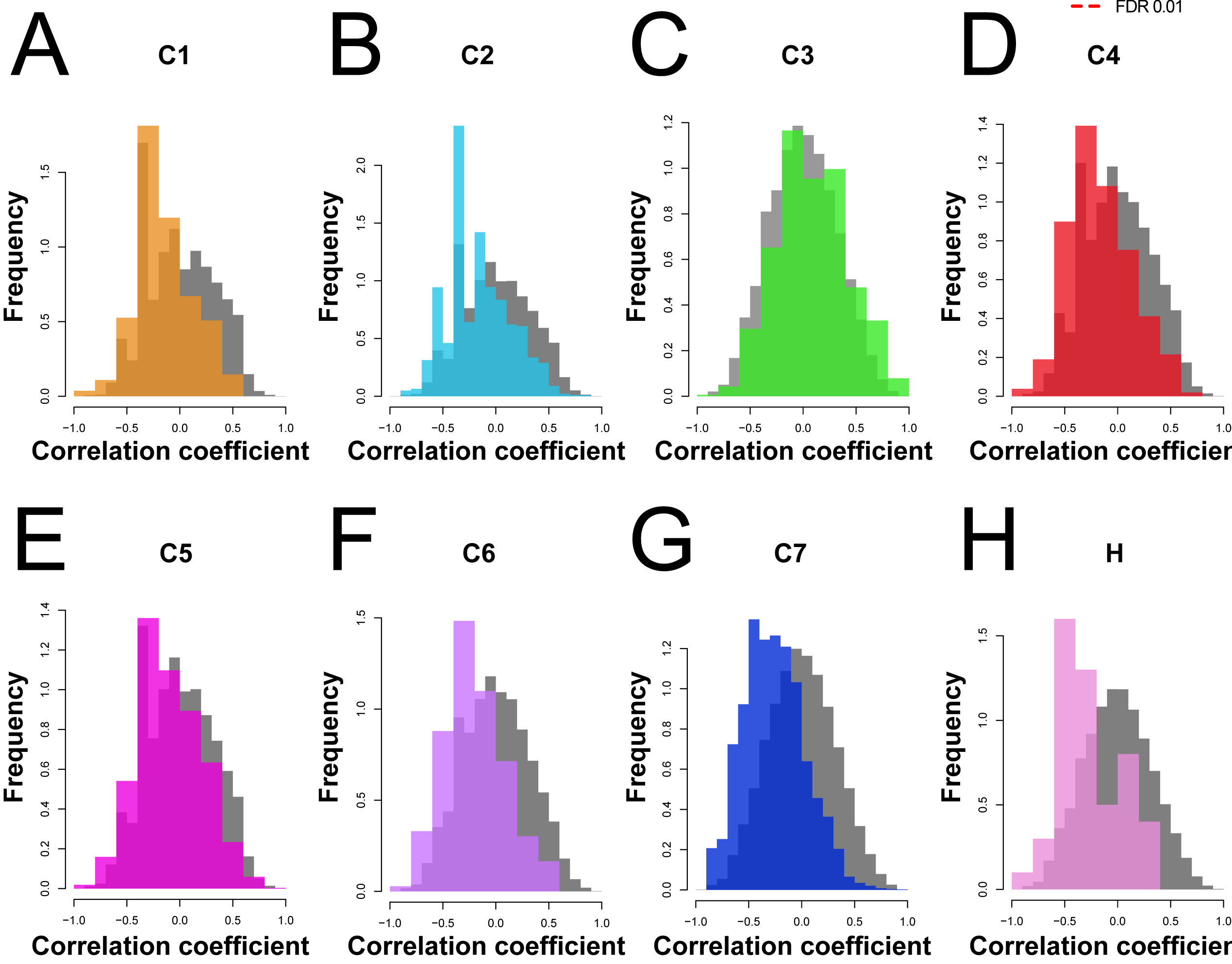
**

**Fig S5 Functional convergence correlation/trend distributions for all MSigDB collections.**  We observe little to no enrichment over the null in the MolSigDB collection gene sets: (A) C1- positional gene sets, (B) C2- curated gene sets, (C) C3- motif gene sets, (D) C4- computational gene sets, (E) C5- GO gene sets, (F) C6- oncogenic signatures, (G) C7- immunologic signatures, and (H) H- hallmark gene sets. However, in a few of the collections, we see negative tails.

### Supplementary Tables

**[See Additional file 2 - excel spreadsheet]**

**Table S1 Functional convergences and correlations/trends**

**Table S2 Additional expression functional convergences and correlations/trends**

**Table S3 Additional gene properties functional convergences and correlations/trends**

**Table S4 Additional MSigDB functional convergences and correlations/trends**

**Table S5 Additional network connectivity functional convergences and correlations/trends**

### Supplementary Data

All gene sets as R binary files, documentation and scripts are available at: <https://github.com/sarbal/EffectSize>
